# Roles and responsibilities of nurses in mechanical ventilation in pediatric intensive care units: a national survey in Austria

**DOI:** 10.1186/s12912-026-04337-0

**Published:** 2026-01-23

**Authors:** Lydia Bauernfeind, Fritz Sterr, Dieter Furthner, Anna Maria Dieplinger

**Affiliations:** 1 Deggendorf Institute of Technology (DIT), Faculty of Applied Healthcare Sciences, Land-Au 27, 94469 Deggendorf, Germany; 2 Paracelsus Medical University (PMU), Institute of Nursing Science and Practice, Salzburg, Austria; 3 Witten/Herdecke University (UW/H), Faculty of Health, School of Nursing Science, Witten, Germany; 4 Salzkammergut hospital Vöcklabruck, Department of Pediatrics and Adolescent Medicine, Vöcklabruck, Austria

**Keywords:** Austria, Cross-sectional study, Mechanical ventilation, Nurse-led weaning, Pediatric intensivists, Pediatric intensive care unit, Roles and responsibilities, Ventilator weaning

## Abstract

**Background:**

In pediatric intensive care units (PICU), mechanical ventilation (MV) and ventilator weaning are common but complex interventions. Internationally, nurses have a central role in ventilator weaning, and the evidence on nurse-led weaning indicates positive effects on the duration of MV. However, little is known about the roles and responsibilities of nurses in Austrian PICUs. This study aims to determine nurses´ roles and responsibilities during MV treatment in Austrian PICUs.

**Methods:**

A cross-sectional study was conducted between May and October 2023. To this end, nurses and physicians from all PICUs in Austria (*n* = 10) completed an online survey with self-developed questionnaires, asking about the use of guidelines, the role in ventilator and sedation weaning and the need for further education. Statistical analysis with SPSS version 29.0 included multiple-response analysis, Pearson´s chi-square test, phi coefficient, Fisher´s exact test, Mann-Whitney-U-test, biserial rank correlation, and Spearman correlation.

**Results:**

107 nurses and 20 physicians responded to the questionnaire. The use of a protocol for MV was reported by 8.1% and for weaning by 1.6% of the participants. Nurses and physicians share responsibility for adjusting the MV settings and assessing weaning readiness. Individual parameters such as oxygen saturation (98.7%), PEEP (30.3%), respiratory rate (50%), pressure control level (42.1%), or ventilation mode (35.5%) are set independently by the nurses. The autonomy of nurses in weaning correlates with increasing professional experience (*r* = 0.318, *p* = 0.008) and the completion of special training for pediatric intensive care (*r* = 0.378, *p* = 0.001). Safety in dealing with mechanically ventilated children also correlates with increasing professional experience in the PICU (*r* = 0.252, *p* = 0.035) and special training for pediatric intensive care (*r* = 0.401, *p* < 0.001).

**Conclusion:**

The results of this survey on the roles and responsibilities of nurses in Austrian PICUs show that protocols for mechanical ventilation and ventilator weaning are rarely used and important decisions are made together by physicians and nurses. The results provide important information for planning future intervention studies to implement a standardized approach based on evidence-based protocols and for developing a special training for Respiratory Specialized Nurses in Austria in line with international standards.

**Trial registration:**

Open Science Framework, 7UTYX, April 18th ,2025. This study was registered retrospectively: 10.17605/OSF.IO/7UTYX.

## Background

Working in a pediatric intensive care unit (PICU) requires knowledge and skills in treating multiple organ dysfunctions, including mechanical ventilation (MV) [1]. MV is a common, but complex intervention to treat a variety of causes of acute respiratory failure in PICUs [2]. As prolonged MV increases the risk of ventilator-associated complications, and is associated with prolonged ICU stay, higher costs, and numerous other risks [3–9], weaning from MV should be considered as early as possible [10].

Determining weaning readiness is highly individual in pediatric patients [11]. During MV, it is therefore essential to continuously monitor and evaluate the patient´s condition to determine the right time to transition from complete MV to ventilator weaning. Clinical factors such as the level of consciousness and sedation, the ability to perform airway clearance by coughing, and the absence of excessive secretions should determine the timing of weaning initiation [12]. The entire process of weaning from MV and the endotracheal tube takes on average 40–50% of th total duration of invasive ventilation [13, 14].

Internationally, nurses play a central role in ventilator weaning, they assess patients and their responses to treatment. Accordingly, evidence on nurse-led weaning indicates positive effects on the duration of MV [15]. In recent years, the implementation of guidelines to support nursing decision-making regarding MV has shortened the time of MV and improved patient survival [16, 17]. Nurses are therefore ideally positioned to recognize weaning readiness and actively participate in the weaning process of MV. Therefore, a greater contribution from nurses in the treatment of mechanically ventilated children would be expected, but this is currently not occurring in Austria. The lack of legal frameworks also makes it difficult to implement nurse-led interventions during MV treatment successfully [18].

To implement such guidelines, the contextual factors need to be considered, and the implementation must be evaluated through clinical trials. However, the effectiveness of randomized controlled trials (RCTs) depends on their ability to function in a closed system, in which the intervention is the only causal force acting on the experimental groups, while it is not present in the control group [19]. This enables a valid assessment of intervention effectiveness. Conversely, clinical settings are open systems in which factors such as context, resources, interpretations, and actions of individuals influence the implementation process and the effectiveness of the interventions. As a result, it can be difficult to define a benchmark (often referred to as ´usual care´) and test it in (multicenter) RCTs. Results are therefore not understandable without consideration of ´usual care´ factors that may influence the implementation process and impact on interventions. For this reason, the MRC Framework [20] describes the need to present ´usual care´ before conducting an RCT or intervention study.

To date, little is known about the ´usual care´, the roles of nurses during MV treatment in Austrian PICUs, and their responsibility for important decisions such as the selection and adjustment of ventilator settings, patient readiness to wean from MV, weaning methods, readiness for extubation, or the recognition of weaning failure. In this regard, the main objective of this study is to determine nurses´ roles and responsibilities during MV treatment in Austrian PICUs.

The secondary objective is to describe influencing contextual factors, including the use of ventilator and weaning protocols, nurses´ perceived autonomy and influence on respirator decisions, satisfaction with current practice, and the need to provide further education on MV.

## Methods

### Design and setting

We conducted a cross-sectional study to depict an overview of the current situation in Austria. The study is reported following the STROBE recommendations for reporting of cross-sectional studies [21].

To avoid selection bias due to the small population of PICU nurses and physicians, we aimed to conduct a survey of all PICUs in Austria (*n* = 10) that care for mechanically ventilated children. In every institution, one nursing and one medical contact person was appointed, who sent the link to the online survey to the staff via their internal mailing list and informed them of the opportunity to participate. Nurses with at least three years of education and physicians (residents and consultants) from all 10 PICUs in Austria who care for children requiring invasive ventilation were included. Nursing assistants and other professions with only short training were consecutively excluded.

### Survey instrument

A validated questionnaire to assess the roles and responsibilities of nurses in mechanically ventilated adult patients in Australia and New Zealand could be identified [22]. This questionnaire has also been used in an adapted form in the United Kingdom for pediatric intensive care patients [23]. As the legislation, education systems, and skills of nurses in these countries are not comparable to those in Austria, the questionnaire could not be used. However, we developed our two questionnaires (one for nurses, one for physicians) on their basis. This process is reported elsewhere [24].

Both questionnaires had six topics, with the questionnaire for nurses comprising a total of 36 questions and the questionnaire for physicians a total of 34 questions. These were mainly closed questions with predefined answers and hybrid questions if the range of possible answers was not fully known. The answer options were either developed from available literature (e.g., which scales/guidelines are used) or were 5-point Likert scales.

The first topic contained questions about the PICU, asking about the use of a guideline for the management of MV and the number of beds and treatment places with MV. The second topic of the questionnaires focused on the weaning of MV. Respondents were asked about the standardized implementation of ventilator weaning, making changes to ventilator settings, and the various knowledge and skills of nurses. Topic three comprised questions on sedation weaning during MV. The fourth topic dealt with the basis of trust between physicians and nurses. In the fifth topic, further competencies of the respondents and their perception of the need for further training in the field of MV were surveyed. Finally, socio-demographic data was collected in the sixth topic. In addition, questions were asked about age, professional experience and already completed additional training.

The questionnaires were developed in German language. Following the steps by Burns et al. [25], a pretest and a pilot test were carried out to assess the clinical sensitivity and thus the completeness, clarity and face validity of the questionnaires.

### Ethical consideration

Research involving human participants is subject to ethical standards that ensure respect for all participants and protect their rights. Following the Declaration of Helsinki, various aspects of research ethics were considered in this study.

First, the study was reviewed and approved by the Salzburg Ethics Committee (Nr. 415-E/2594/50/1-2023). Second, participation in the survey was voluntary, and no participants under the age of 18 were included in the study. The participating nurses and physicians were sent a detailed information letter and a data protection declaration with all their rights (e.g., right of access, right to erasure, right to object to processing, etc.). Informed consent was confirmed by ticking a box before starting the online survey. Since the questions primarily concerned current responsibilities in the work of the interviewees’ professions, the risk of harm or burden to the participants was considered low in relation to the high benefits for the research. Third, the data collected was anonymized, and no conclusions could be drawn about the participants based on the personal data provided (age, professional experience, and completed education).

### Data collection

The data was collected between May and October 2023. The link to the online survey was sent to the contact person at the clinics, and confirmation of forwarding to their employees was requested. After four and eight weeks, the contact persons were asked to send their employees a reminder about the opportunity to participate and to confirm again that they had forwarded the link.

### Data analysis

The collected data was first checked for completeness and consistency before being exported to SPSS version 29.0.2.0 for further analysis. The absolute and relative frequencies were determined, and in the case of ordinal data, the median and range were calculated. For questions with several answers to choose from, a multiple-response analysis was carried out, and the frequencies of the responses were determined. The nominal scaled variables were tested using Pearson´s chi-square test. The phi coefficient was used to determine the effect size for 2 × 2 cross-tabulations. The effect size was assessed with values from 0.1 (small effect), 0.3 (medium effect), and 0.5 (large effect) [26]. If at least one of the expected cell frequencies was under five, Fisher´s exact test was used [27]. To show differences between two groups, two independent samples (nurses and physicians) were tested for differences using the Mann-Whitney U-test. The biserial rank correlation was used to test a correlation between an ordinal and a dichotomous variable, and the Spearman correlation was used to show a correlation between ordinal variables. A p-value of < 0.05 was considered statistically significant [28].

## Results

To determine the statistical population, the number of employees in the *n* = 10 PICUs was surveyed, resulting in a total of *N* = 294 nurses and *N* = 52 physicians. The response rate was 36.4% (*n* = 107) for nurses and 38.5% (*n* = 20) for physicians. The mean professional experience in the PICU was 14 years (SD 10,27) for nurses, and 70.8% had already completed a special education in pediatric intensive care. The physicians had an average of ten years´ professional experience (SD 7.93). The median number of beds in the PICU was eight (min-max [5–20]), with a nurse-patient ratio of 1:2 (70%) for mechanically ventilated children. For patients without MV, a patient-to-nurse ratio of 1:2 was also reported in 54.5% and 1:3 in 27.3%, with varying ratios up to 1:6 reported, particularly during night shifts or in the event of staff and/or resource shortages.

### Use of protocols in Austrian PICUs

The use of protocols for the management of MV was stated by 8.1% of participants, with these being self-developed ventilator protocols or internal guidelines. The existence of a protocol for standardized ventilator weaning was completely denied. Around half of the respondents (47.2%) reported that before extubation, a spontaneous breathing trial is conducted as part of the weaning process. For sedation weaning during MV, 36.8% reported using an assessment and 68.3% stated using an assessment tool to assess withdrawal symptoms.

### Roles and responsibilities of nurses during mechanical ventilation

The assessment of patient response to MV and titration of ventilator settings is largely seen as a joint task between physicians and nurses (85.2%, *n* = 75). Concerning this distribution of tasks, the Mann-Whitney-U-test showed no difference between the professional groups (*p* = 0.465). The remaining 14.8% understand the adjustment of ventilation settings as a duty of physicians and not an autonomous task of nurses. The assessment of whether patients are ready to start ventilator weaning was considered a joint task of nurses and physicians by 76.6%. The remaining 23.4% see it as the responsibility of physicians. Nurses also make no autonomous decisions to start the weaning process. The Mann-Whitney-U-test showed no statistical differences between the professional groups (U = 325, Z=-0.5, *p* = 0.66).

On a scale of 0 *(= no involvement)* to 5 *(= deciding almost everything)*, the autonomy of nurses and the influence on decisions regarding MV were rated as ´medium´ (x̃=3), with a statistically significant, moderate correlation between higher autonomy and increasing professional experience (*r* = 0.318, *p* = 0.008), as well as a moderate effect with already completed special training in pediatric intensive care (*r* = 0.378, *p* = 0.001).

A multiple-response analysis was conducted to investigate which parameters are currently being adjusted independently by PICU nurses in Austria. The frequencies of responses are shown in Table 1. The most frequently mentioned parameter was oxygen concentration, which is adjusted independently by 98.7%. The nurses also stated that all parameters on the ventilator are adjusted when patient-specific, individual limits are discussed and defined by physicians. 42.1% of nurses stated that they adjust pressure levels independently. A chi-square test revealed a difference in the views of physicians, as they stated in 78.6%, that the pressure level can be adjusted independently by nurses (χ2 = 4.74, *p* = 0.03, φ = 0.19). For all other ventilation parameters, there were no significant differences in the view between nurses and physicians.

**Table 1:** Percentage of independent adjustments by nurses

Ventilator parameters	% of independent adjustments by nurses
Oxygen concentration	98.7%
Respiratory rate	50.0%
Pressure level	42.1%
Change of ventilator mode	35.5%
Positive end-expiratory pressure (PEEP)	30.3%

The decision as to whether patients are ready for extubation is seen by both professional groups as a joint decision in 83.5% of cases, with no significant difference (U = 526, Z=-0.22, *p* = 1.0). The self-assessed frequency of nurses´ influence on decisions regarding MV was a median of x̃=4 on a five-point Likert scale, which corresponds to the operationalized variable ´often´. A Mann-Whitney-U-test showed no significant difference between nurses and physicians on the perceived influence of nurses´ views on decisions regarding MV (U = 394, Z=-0.91, *p* = 0.417).

**Fig. 1:**
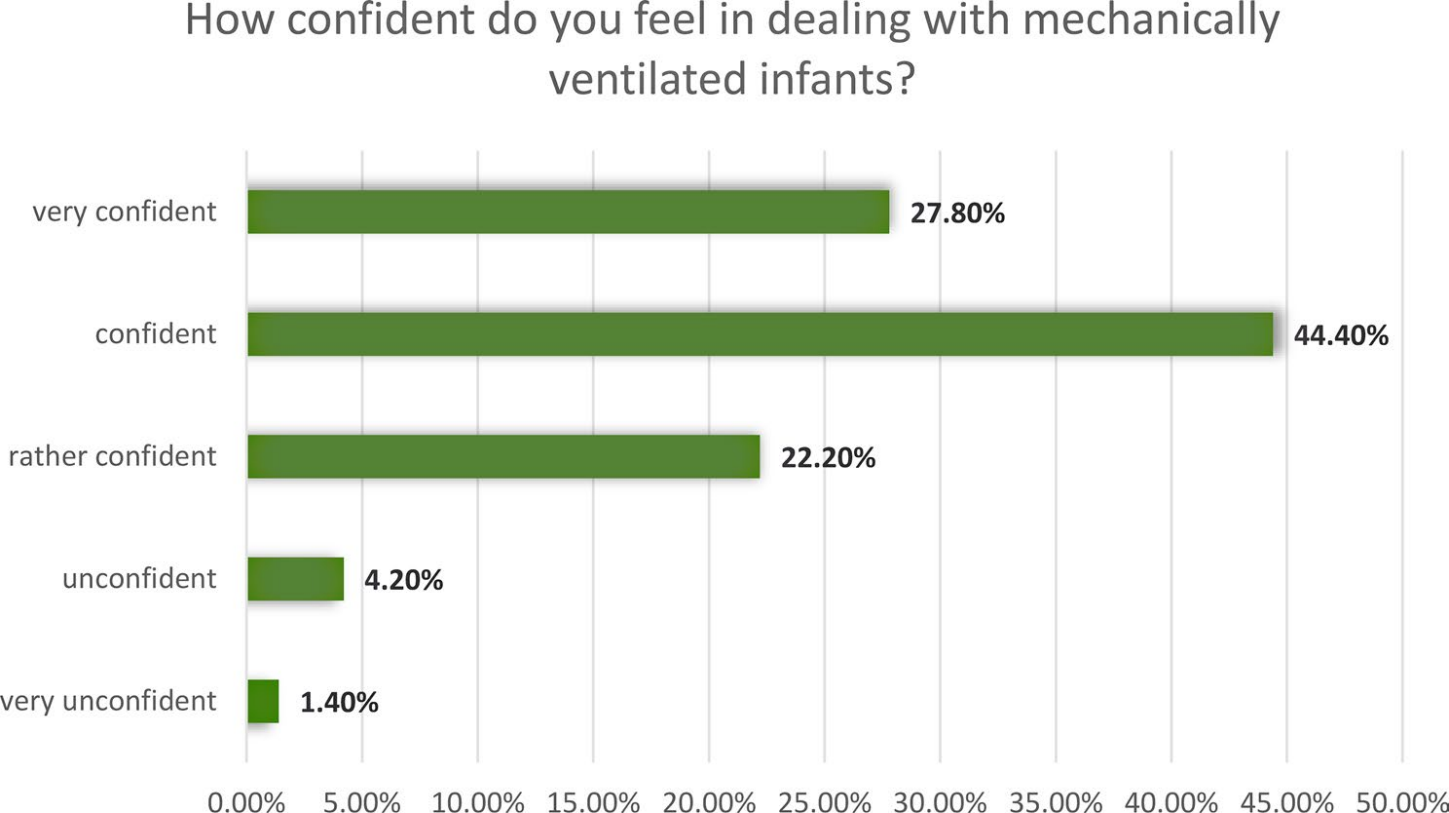
Self-assessed confidence of nurses (*n* = 107) in handling mechanically ventilated children

On a five-point Likert scale, the median self-assessed safety of nurses in handling MV in children was x̃=4, which corresponds to the operationalized variable ´safe´. There was a significant, small correlation between years of professional experience in the PICU and increasing confidence in dealing with invasively ventilated children (*r* = 0.252, *p* = 0.035), as well as a significant, moderate correlation in higher confidence when nurses had already completed the special training for pediatric intensive care (*r* = 0.401, *p* < 0.001). Figure 1 shows the percentage distribution of self-assessed confidence.

The participating physicians were asked whether they would be willing and find it helpful if individual limits were defined for each patient in a nurse-led, standardized protocol so that nurses could adapt ventilator settings independently within these limits. All participating physicians agreed that they trust the nurses both in the clinical assessment and in responding appropriately to changes in patients´ clinical condition. Furthermore, 92.3% trust the nurses in the handling of the ventilator, and 66.7% in the adequate adaptation of ventilator settings.

### Satisfaction with the process of ventilator weaning in Austrian PICUs

The median satisfaction ratings on the five-point Likert scale were x̃=3, which corresponds to the operationalized variable ´rather satisfied´. A Mann-Whitney U-test showed no difference in satisfaction between the two professional groups (U = 399, Z = 0.523, *p* = 0.605). Table 2 shows the percentage of nurses´ and physicians´ satisfaction ratings.

**Table 2:** Satisfaction from nurses and physicians with the process of ventilator weaning in Austrian PICUs

	Physicians (*n* = 20)	Nurses (*n* = 107)
Very unsatisfied	0%	4.1%
unsatisfied	8.3%	13.6%
Rather satisfied	50.0%	38.5%
Satisfied	25.0%	38.5%
Very satisfied	16.7%	5.4%

Participants were also asked about the factors that would influence the satisfaction level in an ideal setting. The frequencies of responses were determined using multiple response analysis. The good cooperation between nurses and physicians was the most frequently selected influencing factor (84.4%), followed by adequate time resources (68.8%) and sufficient knowledge of ventilator modes (66.2%). In addition, participants described adequate training (64.9%), good working conditions (55.8%), standardized procedures for ventilator weaning (42.9%), and good cooperation within the nursing team (32.5%) as key influencing factors for satisfaction with the weaning process.

### Further education for respiratory care

Only 18.3% of participating nurses reported that they had completed additional training in the field of MV. However, 92.3% of physicians and 94.3% of nurses see a high relevance and need for additional training for nurses to expand their knowledge and skills in the field of MV to promote evidence-based care and ensure patient safety. In the questionnaire, there was an optional opportunity to indicate the essential content of a specialized respiratory care training for nurses. This open-ended question was answered by *n* = 40 participants. The responses were categorized, and a multiple-response analysis was conducted to determine the frequency of mentions. The created categories and the frequencies are shown in Table 3.

**Table 3:** Required contents of special education for respiratory care

Categories for education content	Percentage of participants
Basics of MV (including ventilation parameters/ventilation modes)	80.0%
Protocol-based, standardized procedures	40.0%
Ventilator weaning	37.5%
Pharmacology (focus on sedation)	27.5%
Anatomical peculiarities in children	17.5%
Basics of anatomy and physiology	15.0%
Problems and challenges in mechanically ventilated patients	12.5%
Environmental influences on mechanically ventilated children (e.g., noise, light, presence of parents)	12.5%
Simulation-based learning	10.0%
Pathology (focus on diseases requiring MV)	10.0%
Prophylaxis (e.g., ventilator-associated pneumonia)	7.5%
(enteral) Nutrition in mechanically ventilated patients	2.5%
Special hygiene measures	2.5%

## Discussion

To the best of our knowledge, this is the first study to present the current roles and responsibilities of nurses in MV and ventilator weaning of children in Austrian PICUs, as well as influencing contextual factors. To collect the data, we conducted a cross-sectional study with 107 nurses and 20 physicians answering the survey. The results show that protocols are rarely used for MV and weaning from MV in Austrian PICUs. Physicians and nurses predominantly view it as a joint task to adjust MV settings and assess weaning readiness. The autonomy of nurses in weaning and the confidence in dealing with mechanically ventilated children correlate with increasing professional experience and the completion of specialized training in pediatric intensive care.

Due to the multidimensionality and the numerous interacting components required in the care of mechanically ventilated children, defining a comparison group can be challenging [19]. Existing studies on implementing nurse-led, standardized ventilator weaning protocols [16, 29–31] often use ´usual care´ for comparison. However, results from RCTs or intervention studies are difficult to interpret without knowing the factors of usual care. Due to the lack of data in Austria, it was necessary to survey the current roles and responsibilities regarding MV and ventilator weaning of children in PICUs before starting the implementation process for nurse-led, standardized weaning from MV.

Numerous studies [10, 32, 33] recommend a standardized approach in the care of mechanically ventilated children, including ongoing assessment, and enabing rapid and targeted action. Internationally, the practice in PICUs shows that nurses and physicians discuss strategies and set goals regarding MV together. In existing literature is also described that weaning from MV (regardless of the professional group) is not advisable without a plan and a clear goal [1, 2, 16, 33]. Providing adequate prescriptions and standardized protocols allows nurses to act autonomously within certain limits and avoid delays in ventilator weaning. This is described as an essential step to improve the process [34]. However, as shown by the data collected, there are hardly any protocols or standardized procedures in PICUs in Austria for the care of mechanically ventilated children.

Influencing factors described in existing literature and to be considered include a lack of cooperation and collaboration between different disciplines [10], a lack of consensus regarding ventilator weaning methods [29] a lack of interest, continuity, and collaboration between physicians and nurses (e.g. deficiency of setting goals) [2, 35], and a lack of time [1, 36]. Some of the identified barriers are also consistent with the data collected in Austria, as factors like standardization of the process, good cooperation between nurses and physicians, adequate working conditions, additional training opportunities, and sufficient time resources for patients would increase the satisfaction with the process of ventilator weaning.

For adequate management of the ventilator weaning process, continuous monitoring by trained personnel and detailed knowledge of ventilation parameters and their effects on the child´s organism are necessary. However, in clinical practice, ventilator weaning is often started later than actually possible [37]. A possible reason for this is seen in the fact that physicians are responsible for a large number of patients, which leads to a delay in interventions. The survey results confirm this aspect in PICUs in Austria, as physicians reported a staffing level of two to three physicians during dayshifts, and one physician during nightshifts. Nurses are responsible for the care of an average of two patients when MV is required. Due to this staffing situation, the results of this study already highlight that nurses are acting outside of their competencies in the care of mechanically ventilated children. In addition to the skills of nurses, they not only adjust the oxygen concentration, but also ventilator parameters such as PEEP, respiratory rate, and pressure level. Even a complete change of ventilator mode is performed independently by over a third of the nurses surveyed, without a standardized procedure or protocol. However, according to the physicians surveyed, these adjustments can be performed independently by nurses, as they spend significantly more time at the bedside and know the patients better. Furthermore, physicians trust nurses in their clinical assessment of patients, in responding appropriately to changes in clinical condition, in operating the respirators, and in adapting the ventilators appropriately. However, a problem arises that the required skills are acquired through professional experience and/or a lack of physicians’ presence, rather than through additional training and continuing education. To achieve qualitative advancement of professional health and nursing care in PICUs in Austria, it is necessary to define competency and qualification profiles and offer opportunities for specialization. The need for specific knowledge of the (patho)physiology of MV, expertise in the use of weaning support measures, and mastery of various forms and modes of MV are recommended as prerequisites for high-quality care and patient safety [38].

In international comparison, advanced practice nurses are trained at master´s level for the care of mechanically ventilated children and are deployed in clinical practice [39, 40]. A similar survey on roles and responsibilities in mechanically ventilated pediatric patients in the United Kingdom showed that nurses must complete special training in MV right from the start of their work in an ICU. For further specialization, nurses must complete advanced modules on ventilation at master´s level [23]. In Austria, nurses working in PICUs are legally required to complete special training in pediatric intensive care. However, this training only comprises 60 ECTS *(European Credit Transfer System = an instrument used to structure higher education studies in Europe and make the weighting of its components transparent)*, of which only 1 to 2 ECTS (depending on the institution) are for MV, and is therefore not comparable to a master´s level specialization. There are no adequate training programs for the complex intervention of caring for mechanically ventilated pediatric patients in Austria. For the professionalization of health and nursing care in Austria, it is imperative that ‘not everyone does everything’, but that specializations are offered in certain (highly) complex topics, such as MV. Only a small percentage of the nurses surveyed had additional training in the field of MV, and none of the participants mentioned any in-depth training or specialization in this area.

However, the nurses surveyed perceived a high need for further education on MV and ventilator weaning. They also stated that possible training opportunities and the possibility of specialization would be associated with increased job satisfaction. The available data from the survey further indicates that the increasing professional experience of nurses, as well as the availability of advanced training, influence the provision of care. For example, with increasing professional experience and additional training, increased involvement in ventilator weaning of invasively ventilated children was perceived. A similar relationship between a higher level of nursing education and involvement in the weaning process has also been found in existing literature [41].

Due to varying conditions, such as different diseases or the age of the pediatric patients, despite existing standards, physicians and nurses together need to define individual limits for ventilator parameters and other values for each patient. This is the only way to enable autonomous action by nurses according to a protocol. The data collected shows that a large percentage of the physicians surveyed are willing to set these individual limits. Their main argument for promoting autonomous action by nurses and willingness to specify these parameters was nurses’ time spent at patient´s bedside. In addition, physicians report that they trust nurses both in their clinical assessment of patients and in their appropriate response to changes in clinical condition, as well as in the operation of ventilators and the appropriate adjustment of ventilator parameters. These data provide a key point for starting an implementation process. However, adequate preparation and comprehensive educational measures are required before implementation. Furthermore, compliance must be monitored during the implementation process and beyond to prevent the variability among different physicians as well as a lack of interest in implementing nurse-led, standardized weaning from MV.

### Limitations

This study also has limitations. First, due to the faster contact with the hospitals, this was carried out via email or telephone, and the questionnaires were not available in printed form. In this context, the response rate (nurses 36.4% / physicians 38.5%) must also be considered as a limitation. Presenting the research directly at the hospitals or providing printed questionnaires as an alternative option could have potentially increased the response rate. Nevertheless, to make it easier to answer the questionnaire and to avoid the need to actively return it via post, the survey link was distributed exclusively by email. Second, the collected data consists primarily of nominal and ordinal data, with only a few metric data points. Therefore, no conclusion on causal relationships can be drawn. Third, the self-developed questionnaires were tested in a pretest for clinical sensitivity, face validity, and objectivity, but their validity and comparability between different institutions remain limited. Fourth, the participants´ self-reported information and answers may distort actual clinical practice. In addition, the study was registered retrospectively after the survey was completed, which is another noteworthy limitation.

## Conclusion

The results of this study show that protocols for mechanical ventilation and ventilator weaning are rarely used in Austrian PICUs. Key decisions are usually made by physicians and nurses together, with nurses seldom making them autonomously. Due to the lack of data in Austria, it was necessary to record the current roles and responsibilities regarding mechanical ventilation and ventilator weaning of children in PICUs. The results of this survey provide important information for planning future intervention studies to implement a standardized approach based on protocols and for developing a special training for Respiratory Specialized Nurses in line with international standards as high-quality care for mechanically ventilated children can only be guaranteed through adequate academic education and the successful implementation of evidence-based interventions.

## Data Availability

Abstract with poster presentation: Congress of the International Council of Nurses, 12 th June 2025, Helsinki, Finland. Questionnaire-Development: DOI: 10.1007/s16024-024-00417-w. Questionnaire (German language): available from the corresponding author on reasonable request. Dataset: The datasets used during the current study are available from the corresponding author on reasonable request.

## Abbreviations

ECTS: European Credit Transfer System
ICU: Intensive Care Unit
MRC: Medical Research Council
MV: Mechanical Ventilation
PEEP: Positive endexpiratory pressure
PICU: Pediatric Intensive Care Unit
RCT: Randomized Controlled Trial
